# Comparison of different sampling techniques and of different culture methods for detection of group B streptococcus carriage in pregnant women

**DOI:** 10.1186/1471-2334-10-285

**Published:** 2010-09-29

**Authors:** Nabil A El Aila, Inge Tency, Geert Claeys, Bart Saerens, Piet Cools, Hans Verstraelen, Marleen Temmerman, Rita Verhelst, Mario Vaneechoutte

**Affiliations:** 1 Laboratory Bacteriology Research, Department of Clinical Chemistry, Microbiology & Immunology, University of Ghent, Ghent, Belgium; 2 Department of Obstetrics & Gynaecology, Ghent University Hospital, University of Ghent, Ghent, Belgium; 3 Laboratory for Clinical Biology, Ghent University Hospital, University of Ghent, Ghent, Belgium

## Abstract

**Background:**

*Streptococcus agalactiae *(group B streptococcus; GBS) is a significant cause of perinatal and neonatal infections worldwide. To detect GBS colonization in pregnant women, the CDC recommends isolation of the bacterium from vaginal and anorectal swab samples by growth in a selective enrichment medium, such as Lim broth (Todd-Hewitt broth supplemented with selective antibiotics), followed by subculture on sheep blood agar. However, this procedure may require 48 h to complete. We compared different sampling and culture techniques for the detection of GBS.

**Methods:**

A total of 300 swabs was taken from 100 pregnant women at 35-37 weeks of gestation. For each subject, one rectovaginal, one vaginal and one rectal ESwab were collected. Plating onto Columbia CNA agar (CNA), group B streptococcus differential agar (GBSDA) (Granada Medium) and chromID Strepto B agar (CA), with and without Lim broth enrichment, were compared. The isolates were confirmed as S. agalactiae using the CAMP test on blood agar and by molecular identification with tDNA-PCR or by 16S rRNA gene sequence determination.

**Results:**

The overall GBS colonization rate was 22%. GBS positivity for rectovaginal sampling (100%) was significantly higher than detection on the basis of vaginal sampling (50%), but not significantly higher than for rectal sampling (82%). Direct plating of the rectovaginal swab on CNA, GBSDA and CA resulted in detection of 59, 91 and 95% of the carriers, respectively, whereas subculturing of Lim broth yielded 77, 95 and 100% positivity, respectively. Lim broth enrichment enabled the detection of only one additional GBS positive subject. There was no significant difference between GBSDA and CA, whereas both were more sensitive than CNA. Direct culture onto GBSDA or CA (91 and 95%) detected more carriers than Lim broth enrichment and subculture onto CNA (77%). One false negative isolate was observed on GBSDA, and three false positives on CA.

**Conclusions:**

In conclusion, rectovaginal sampling increased the number GBS positive women detected, compared to vaginal and/or rectal sampling. Direct plating on CA and/or GBSDA provided rapid detection of GBS that was at least as sensitive and specific as the CDC recommended method of Lim broth subcultured onto non chromogenic agar.

## Background

*Streptococcus agalactiae *(group B streptococcus, GBS) is a significant cause of perinatal and neonatal infections worldwide. Rectovaginal colonization occurs in 10 to 30% of pregnant women [1-3] and is responsible for 1.8 neonatal infections per 1,000 live births per year [4]. In Belgium, 13 to 25% of pregnant women are colonized with GBS. GBS is responsible for 38% of early neonatal infections [5].

GBS can be acquired during labor or in utero by transmission from maternal vaginal or anorectal-colonized mucosa. Prematurity is also a risk factor for GBS neonatal sepsis, and mortality due to GBS is higher in preterm than in term newborns [6]. Because results at 35-37 weeks correlate more closely with GBS colonization at term delivery, the Centers for Disease Control and Prevention (CDC) has recommended that all pregnant women be screened for carriage of GBS at between 35 and 37 weeks of gestation [7], so that GBS positive women can receive antibacterial treatment (chemoprophylaxis) prior to delivery, to reduce mother-to-child transmission.

To maximize GBS carriage detection rates, both the anatomic site of sampling and the culture methods used are important. Rectovaginal swabs have been reported to provide high bacterial yields, as the gastrointestinal tract is a natural reservoir for GBS and a potential source of vaginal colonization [7-11]. In the present study, we compared three sampling techniques, i.e. rectovaginal swabbing, vaginal swabbing only and rectal swabbing only, using the ESwab, a nylon flocked swab in liquid Amies transport medium (Copan, Brescia, Italy), which, according to the manufacturer, is a liquid-based multipurpose collection and transport system that maintains viability of aerobic, anaerobic and fastidious bacteria for up to 48 hours at room and refrigerator temperature, and suitable for automation, gram stains, and traditional culture, and which compares well to other swabs with regard to species recovery [12,13].

With regard to bacterial culture, the CDC recommends isolation of the bacterium from vaginal and rectal swabs by growth in a selective enrichment medium, such as Lim broth, i.e. Todd-Hewitt broth with colistin and nalidixic acid, followed by subculture on sheep blood agar. In the same guidelines, the CDC identified various research priorities, including 'the development of media with a reliable color indicator to signal the presence of GBS to improve accuracy of prenatal culture results and facilitate prenatal culture processing at clinical laboratories with limited technical capacity' [14].

Therefore, in the present study, we also determined the sensitivity and specificity of two types of color indicator based media that are commercially available for detecting GBS carriage in pregnant women, with that of the CDC recommended method, i.e. Lim broth enrichment with subculture onto sheep blood agar.

The first type, designated Granada Medium (GM) [15], is an adaptation of Islam's medium [16], and exploits the ability of GBS to synthesize - under anaerobic conditions and on media containing starch and serum - an orange pigment, recently identified as granadaene [17].

This method is very specific and simple, thereby allowing identification of GBS in a single step within 24 h. Later, a modification of GM was described as new GM [15]. In this study, we used group B streptococcus differential agar (GBSDA, Becton Dickinson), which is itself a modification of new GM and of which the manufacturer claims that it has improved selectivity and stability compared to new GM, without further specification. The second type of medium used in this study, recently developed by bioMérieux as chromID™ Strepto B agar (CA), is a selective chromogenic medium, of which the constituents are not specified by the company, and which enables the recognition of *S. agalactiae *as pink to red, round and pearly colonies, without the need of anaerobic incubation. Most other bacterial species are either inhibited or the colonies produced have a different colour (e.g. violet, blue, colourless) [18-20].

In summary, in addition to comparing three sampling methods, we compared six different culture methods, i.e. direct culture onto Columbia Colistine Nalidixic Acid Agar (CNA), GBSDA and CA, and Lim broth enrichment with subculture on these three agars.

## Methods

### Study design

The study was approved by the research ethics committee (IRB protocol nr 2007/096) of the Ghent University Hospital, Flanders, Belgium, and all the women gave written informed consent. Between June 2009 and January 2010, 100 vaginal samples, 100 rectal and 100 rectovaginal ESwab samples were collected from 100 pregnant women at 35 - 37 weeks of gestation, i.e. three different samples per subject.

### Collection and culture of specimens

Rectovaginal, vaginal and rectal samples were collected using nylon flocked swabs that were submerged into 1 ml of ESwab transport medium (ESwab, Copan Diagnostics, Brescia, Italy).

Rectovaginal sampling was carried out by rotating an ESwab against the vaginal wall at the midportion of the vault. Subsequently, the swab was carefully withdrawn to prevent contamination with microflora from the vulva and introitus and the swab was inserted 1.5 to 2 cm beyond the anal sphincter and gently rotated to touch the anal crypts. Next, vaginal sampling was carried out by inserting the ESwab following the same procedure described above for swabbing the vaginal wall. Finally, an ESwab was used for rectal sampling as described above for the anal procedure of the rectovaginal sampling.

**Figure 1:**
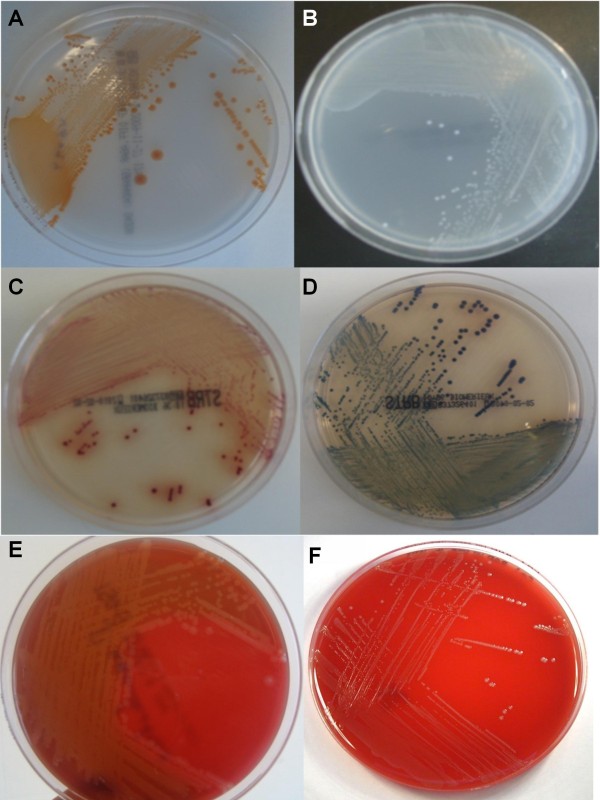
**Appearance after 24 h incubation of (A): *Streptococcus agalactiae *(Group B *Streptococcus *(GBS) and (B) *Enterococcus faecalis *on GBSDA, (C) GBS and (D) *Enterococcus faecalis *on Strepto B ID^® ^chromogenic agar and (E) GBS and (F) *Enterococcus faecalis *on CNA**.

All samples were collected by midwives and transported to the Laboratory of Bacteriology Research within 4 hours. Direct plating was carried out only for the rectovaginal ESwab, by inoculating 50 μl from the ESwab transport medium onto Columbia agar with 5% sheep blood and with 10 mg/ml colistin and 15 mg/ml nalidixic acid (CNA, Becton Dickinson, Erembodegem, Belgium), 50 μl onto group B streptococcus differential agar (GBSDA, Becton Dickinson) and 50 μl onto chromID™ Strepto B agar (CA, BioMérieux, Marcy l'Etoile, France). The CNA plates were incubated at 37°C in 5% CO_2 _for 24-48 h, the GBSDA plates were incubated at 37°C in an anaerobic chamber (BugBox, LedTechno, Heusden-Zolder, Belgium) for 24-48 h, and the CA plates were incubated at 37°C for 18-24 hours in aerobic conditions in the dark. Volumes of 200 μl from the ESwab transport medium of the rectovaginal, vaginal and rectal ESwabs were inoculated into separate tubes with 5 ml of Todd-Hewitt broth with 1% yeast extract, 15 μg/ml nalidixic acid and 10 μg colistin/ml (Lim broth, Becton Dickinson), which were incubated aerobically at 37°C and subcultured onto CNA, GBSDA and CA after overnight incubation. GBSDA was examined for yellow-orange pigment colonies indicative of the presence of GBS, whereas CA was examined for pale pink to red, round and pearly colonies. β-haemolytic and non-haemolytic colonies were picked from CNA for further identification (Figure 1). The isolates were confirmed as *S. agalactiae *using the CAMP test on sheep blood agar. GBS colonies with discrepant results (either false positive on CA or false negative on GBSDA) were identified using tDNA-PCR, as described previously [21], and by 16S rRNA gene sequence determination.

### Statistical methods

The McNemar test for correlated percentages was used to compare the sensitivity of the culture methods. The total number of positive subjects (22) was taken as 100%, to calculate sensitivities, specificities, positive and negative predictive values of the different sampling and culture methods.

## Results

A total of 300 swabs was taken from 100 pregnant women at 35-37 weeks of gestation, and 49 of these swabs were positive for a total of 22 subjects. Data for each subject, sampling site and method and culture method are listed in additional file 1, Table 1. GBS could be cultured from all 22 rectovaginal swabs, although one was only positive after Lim broth enrichment. Of the 22 GBS positive subjects, GBS could be cultured for nine from both the rectal and vaginal swabs, for two only from the vaginal swab, and for nine exclusively from the rectal swabs. For another two women, only the rectovaginal swab was positive.

**Table 1:** Number of GBS culture positive samples detected by different culture media in rectovaginal, vaginal and rectal specimens obtained from 49 GBS positive samples from 22 GBS positive women

Specimen	CNA	GBSDA	CA	Lim-CNA	Lim- GBSDA	Lim- CA	Estimated number of women colonized
**Rectovaginal ESwab**	13	20	21	17	21	22	22
**Vaginal ESwab**	NT	NT	NT	9	11	11	11
**Rectal Eswab**	NT	NT	NT	13	17	18	18

CA: Chrom ID Strepto B agar (BioMérieux), CNA: colistin nalidixic acid Columbia agar, GBSDA: GBS Differential Agar (Becton Dickinson), Lim: Lim broth.

NT: Not tested.

The GBS detection rate on the basis of rectovaginal samples (22 GBS positive women) were significantly higher than the detection rate on the basis of vaginal samples (11 positive) (*P *= 0.01), but not significantly higher than that on the basis of rectal samples (18 positive) (*P *= 0.12).

Direct plating of the rectovaginal swabs on CNA, GBSDA and CA resulted in detection of 59, 91 and 95% respectively of the total number of carriers detected on all samples and media, whereas Lim broth with subculture onto on CNA, GBSDA and CA resulted in positivities of 77, 95 and 100% respectively (Table 1). For all sampling methods, and for the rectovaginal swab with and without enrichment, GBSDA and CA detected more positive women than CNA. In addition, detection of GBS from rectovaginal specimens by direct plating onto GBSDA or CA was equally sensitive as detection by Lim broth enrichment with subculture on these agars (*P *= 1) and only one pregnant woman (RVS001) was identified as GBS-positive only after Lim broth enrichment. It should be noted that the inoculum of GBS in this subject was low, as only a few colonies were observed on the agars, even after the enrichment incubation. One false-negative result on GBSDA, i.e. a colony without orange pigmentation, corresponded to a non-haemolytic and non-pigmented GBS strain that was correctly identified on CA. Three false positives for three different women were observed on CA, because the red colonies isolated from CA were all three identified as *Streptococcus anginosus*, by means of 16S rRNA gene sequence determination and/or tDNA-PCR [21].

## Discussion

This study intended to compare the sensitivity of different sampling and culture procedures to establish the presence of GBS in pregnant women. We compared i) rectovaginal, vaginal and rectal sampling, and ii) culture on Columbia CNA agar (CNA), on group B streptococcus differential agar (GBSDA) and on Chromogenic Strepto B ID Agar (CA), iii) directly and after Lim broth enrichment. Other groups studying chromogenic agar did so only for vaginal samples [18-20,22], whereas we also included the CDC recommended rectovaginal sampling method, or they did not compare to Granada agar and used miscellaneous samples [23].

A limitation of this study may be the limited sample size of one hundred subjects, although it should be noted that each subject was studied intensively, i.e. three different sampling sites and six different culture methods were compared for each subject, in a strictly designed study setup that enabled to compare the culture results for each subject in a direct and unambiguous manner.

### Comparison of rectovaginal, vaginal and rectal sampling

We found that rectovaginal swabbing was the best sampling method to detect GBS colonization of pregnant women, because all 22 GBS positive women in this study were detected by means of rectovaginal sampling and because two subjects were GBS positive only on the basis of the rectovaginal swab. Our results correspond with previous reports that GBS colonization of rectal samples is 18% to 24% higher than that of vaginal samples [24,25] and with those of other studies that find rectovaginal sampling more appropriate than vaginal sampling only [7,26,27]. For example, in an analysis of 651 specimens, the combination of separate rectal and vaginal sampling enabled detection of 97.8% of GBS carriers, compared to 31.8% of positives as established by vaginal sampling only [28]. Although our results are in correspondence with the CDC recommendations to carry out rectovaginal sampling, it should be noticed that Nomura *et al*. [29] found no significant difference in detection rates between vaginal and rectal samples and Gupta & Briski [30] reported a similar detection rate of 23.8% of GBS when using rectovaginal and vaginal sampling. Votava *et al*. [11] even found that the GBS detection rate using rectovaginal samples was only 16.9%, whereas the use of separate vaginal and rectal swabs yielded 22.7 and 24.1% GBS positive women, respectively. Also, several obstetric departments still use vaginal sampling only to assess GBS positivity.

It is worthwhile mentioning that we also compared vaginal sampling using the recently marketed ESwab with vaginal sampling using the classical cotton swab in Amies gel transport medium (Nuova Aptaca, Canelli, Italy) for the detection of GBS (data not presented to increase readability of the manuscript). Both swabs were introduced simultaneously into the vagina and culture was carried out in an identical manner for both swabs. The classical swab could detect two more GBS positive subjects than the ESwab, raising the number of women positive for GBS in the vagina from 11 to 13.

#### Different types of selective media formulations

Different selective media for the improved isolation of GBS have been described. Islam *et al*. [16] showed that adding horse serum and starch to agar based media increased the orange/red pigment formation, that was already present to some degree on Columbia agar and that was typical for GBS, being absent for all other serotypes. De la Rosa *et al*. [31] improved this medium adding horse serum at 90 to 95°C instead of at 55°C, making the agar opaque and increasing the natural pigmentation of the colonies, specific for *S. agalactiae*. They showed that pigmentation depends strongly on the use of the correct starch and of proteose peptone n°3 and that it was further increased by the addition of the folate inhibitor trimethoprim (15 μg/ml), in combination with anaerobic incubation. This medium was designated Granada Medium (GM). The same authors later improved GM, which they designated as New Granada medium (NGM) [15], by replacing trimethoprim with 6 μg/ml methotrexoate, which is also a folate synthesis inhibitor, but further increases orange to salmon pigmentation of the GBS colonies, and by adding 0.2 μg/ml crystal violet, 5 μg/ml colistin sulphate and 10 μg/ml metronidazole as selective agents. It should be noted that horse serum was added again at 55°C. The commercially available group B streptococcus differential agar (GBSDA), that was used in this study, is a modification of NGM with improved stability and selectivity, not further specified by the company. Its usefulness has been evaluated in several studies [9,32-34]. Bou *et al*. [32] found that the intensity of colony pigmentation on GBSDA is stronger than on GM and that the commensal microflora is more suppressed.

Because different modifications have been used, sometimes also designated as GM, or because the media were home made [35] or prepared by other companies than the one that supplied the GBSDA in this study [30,36], or because other selective media were used [8,37,38], it is difficult to compare the outcome of several of the previous studies with this study. Therefore, we largely limit our comparison to studies that explicitly used CA from BioMérieux and/or GBSDA from Becton Dickinson.

#### Direct plating vs broth enrichment culture

Possibly, the use of different (commercial) preparations, modifications and designations, as mentioned above, may explain why some studies found comparable sensitivity of direct plating on 'Granada Medium' compared with Lim broth enrichment [11,15,39,40], whereas other studies found direct plating on chromogenic and/or selective media significantly less sensitive [30,36]. Blanckaert *et al*. [41] suggested to use a combination of Granada and Columbia blood agar and an adequate sample (rectovaginal swab in transport medium) for optimal GBS screening.

In our study, the sensitivity of direct plating on CA and GBSDA was comparable to that of plating on CA and GBSDA after Lim broth enrichment, whereby the latter enabled the detection of only one additional sample, leading to 22 GBS (22%) positive women. Our data suggest that CA and GBSDA are not only faster and easier to use than the CDC recommended method, but that they are also at least as sensitive for the detection of GBS, in agreement with several other recent studies. Also Tazi *et al*. [18] found that, compared to CA and GBSDA, Lim broth enrichment enabled the detection of only two additional samples leading to 34 (17%) GBS positive cultures. Also in the study of Bou *et al*. [28], only one swab was only positive following subculture in Lim broth and was missed on direct GBSDA [32]. Adler *et al*. [34] reported that GBSDA is not only faster and easier to use than Lim broth combined with antigen detection or with subculture on blood agar, but is also at least as sensitive for the detection of GBS from vaginal swabs. In addition, Dunne et al. [8] reported that direct plating on neomycin nalidixic acid agar reduced the potential of enterococci competitively overgrowing and masking the presence of GBS in the Lim broth, and this ultimately increased the sensitivity of the direct assay by 14%.

In conclusion, although we missed one out of 22 carriers by direct plating on CA and GBSDA, in our hands direct plating on CA and GBSDA provided high sensitivity for GBS detection among pregnant women.

#### CA and GBSDA vs colistin nalidixic acid selective agar (CNA)

We found that CA and GBSDA had comparable sensitivity and provided superior recovery of GBS when compared with CNA. This difference was even more apparent for direct plating. Also, the single subject (RVS072) positive only for the rectovaginal swab was so only on CA and GBSDA, but not on CNA. This is in agreement with another study [30] that showed that selective media producing pigmented colonies are more sensitive in GBS detection than enriched media like blood agar or selective media like CNA.

Direct plating on CA and GBSDA offers the advantage of reducing workload and providing an identification of GBS 24 h sooner than the Lim broth enrichment method. In this study, all GBS isolated from CA and GBSDA were identified within 1-2 days of specimen receipt, whereas all Lim broth enrichment cultures required a minimum of 2-3 days for the identification of specimens positive for GBS. In addition, CA offers an additional advantage with respect to GBSDA, because culture on CA can be carried out aerobically, not requiring special equipment and extra costs and workload associated with anaerobic culture needed for GBSDA.

### Sensitivity, specificity, positive and negative predictive value of GBS detection with CA versus GBSDA

The sensitivity and positive predictive value of direct plating of CA for GBS detection were 100 and 87, respectively (Table 2), whereas these values for direct plating on GBSDA were 95 and 100, respectively. In this study, one false negative isolate, lacking the orange pigment, was found on GBSDA, for subject RVS041. This isolate was not missed on CA and was confirmed as GBS by means of the CAMP test. Also Tazi *et al*. [18] found two false negative isolates on Granada medium. Non-haemolytic and non-pigmented GBS have been reported to occur in 1 to 4% among pregnant women [11,30,36,39,40]. Pigment is produced by 93 to 98.5% of GBS clinical isolates. There is a high correlation between the capacity to produce pigment and the capacity to release hemolysin [42,43], since the genes that determine these properties are in contiguous loci on the chromosome [40].

**Table 2:** Sensitivities, specificities, positive and negative predictive values for the six different culture methods, based on all 300 samples, and calculated for a number of 22 positive subjects on a total number of 100 subjects included.

Culture Medium	% Sensitivity	% Specificity	% Positive predictive value	% Negative Predictive value
**CNA**^**a**^	56	84	52	86
**GBSDA**	95	100	100	99
**CA**	100	96	87	100
**Lim broth + CNA**	78	84	60	93
**Lim broth + GBSDA**	96	100	100	99
**Lim broth + CA**	100	96	88	100

a: False positive on CNA: beta-hemolytic colonies which were CAMP negative.

In our study, we found three false positive results with CA after 24 hrs of incubation, whereby all three isolates were identified as *Streptococcus anginosus*. Tazi *et al*. [18] found two false positive results on Chromagar and showed that these corresponded either to *Streptococcus pyogenes *or *S. porcinus*. These observations indicate that colonies that grow on CA and are suspected to be GBS must be confirmed by additional tests such as CAMP, latex agglutination or molecular techniques, or by positivity on GBSDA. It may be suggested that the combination of direct plating onto CA with aerobic incubation (95% sensitivity), with confirmation of CA positive isolates by means of the CAMP test (100% specificity), may be a highly sensitive, specific and cost effective manner to detect GBS from pregnant women.

## Conclusions

In conclusion, to detect GBS carriage among pregnant women, our results indicate i) that rectovaginal sampling is the preferred sampling method, ii) that the ESwab is not superior to the classical cotton swab for sampling, iii) that the inoculation of rectovaginal specimens directly onto CA (21 positives/22) and/or GBSDA (20 positives) has comparable sensitivity as enrichment by Lim broth (22 and 21 positives after subculture onto CA and GBSDA, respectively), and iv) that direct inoculation onto CA or GBSDA is at least as sensitive as the recommended CDC method, i.e. overnight Lim broth enrichment followed by plating onto sheep blood agar (which in this study was replaced by CNA: 17 positives).

Direct inoculation offers several advantages such as decreased workload, because no subculture is needed, and decreased time to detection, i.e. at least 24 hours faster than the standard method. Reagent costs of using CA or GBSDA may be comparable to Lim broth enrichment and subculture on blood agar, also because additional testing is rarely needed for the former approach. Plating on CA in addition does not require anaerobic incubation as is the case for GBSDA. The specificity problems associated with the use of CA can be resolved by confirmation of CA positive isolates with the CAMP test.

## Competing interests

The authors declare that they have no competing interests.

## Authors' contributions

NAE, RV, GC and MV participated in the development of the study design, the analysis of the study samples, the collection, analysis and interpretation of the data, and in the writing of the report. IT, HV and MT participated in the development of the study design, the collection of the study samples, the collection, analysis and interpretation of the data, and in the writing of the report. BS and PC participated in the analysis of the study samples and interpretation of the data. All authors read and approved the final manuscript.

## Pre-publication history

The pre-publication history for this paper can be accessed here:

http://www.biomedcentral.com/1471-2334/10/285/prepub

## Supplementary Material

Additional file 1 **Table 1: Detection of GBS by means of culture on different media for vaginal, rectal and rectovaginal samples from 22 GBS positive pregnant women**. Three samples were collected from pregnant women (vaginal, rectal and rectovaginal). The rectovaginal samples were cultured directly and after Lim broth enrichment on the following media (CNA, GBSDA and CA) whereas vaginal and rectal samples were cultured only after Lim broth enrichment on the same three media. Click here for file
