# Supplementary material for: Comparison of different sampling techniques and of different culture methods for detection of group B streptococcus carriage in pregnant women
Source: BMC Infect Dis. 2010 Sep 29;10:285. doi: 10.1186/1471-2334-10-285 (PMC2956727; doi:10.1186/1471-2334-10-285)
Supplement: Additional file 1 — Table 1: Detection of GBS by means of culture on different media for vaginal, rectal and rectovaginal samples from 22 GBS positive pregnant women. Three samples were collected from pregnant women (vaginal, rectal and rectovaginal). The rectovaginal samples were cultured directly and after Lim broth enrichment on the following media (CNA, GBSDA and CA) whereas vaginal and rectal samples were cultured only after Lim broth enrichment on the same three media. [file 1471-2334-10-285-S1.DOC]

**Additional file 1, Table 1.**

| **Sample** | **VR** | **VR** | **VR** | **VR** | **VR** | **VR** | **V** | **V** | **V** | **R** | **R** | **R** |  |  |
| --- | --- | --- | --- | --- | --- | --- | --- | --- | --- | --- | --- | --- | --- | --- |
| **Lim-broth** | **N** | **N** | **N** | **Y** | **Y** | **Y** | **Y** | **Y** | **Y** | **Y** | **Y** | **Y** |  |  |
| **Agar** | **CNA** | **GBSDA** | **CA** | **CNA** | **GBSDA** | **CA** | **CNA** | **GBSDA** | **CA** | **CNA** | **GBSDA** | **CA** |  |  |
| **Subject** |  |  |  |  |  |  |  |  |  |  |  |  | **N° cultures positive** | **N° samples positive** |
| RVS001 | 0 | 0 | 0 | 0 | 1 | 1 | 0 | 0 | 0 | 0 | 1 | 1 | 4 | 2 |
| RVS003 | 1 | 1 | 1 | 1 | 1 | 1 | 1 | 1 | 1 | 1 | 1 | 1 | 12 | 3 |
| RVS009 | 1 | 1 | 1 | 1 | 1 | 1 | 1 | 1 | 1 | 0 | 1 | 1 | 11 | 3 |
| RVS015 | 0 | 1 | 1 | 1 | 1 | 1 | 1 | 1 | 1 | 1 | 1 | 1 | 11 | 3 |
| RVS018 | 0 | 1 | 1 | 1 | 1 | 1 | 0 | 0 | 0 | 1 | 1 | 1 | 8 | 2 |
| RVS021 | 0 | 1 | 1 | 1 | 1 | 1 | 1 | 1 | 1 | 0 | 0 | 0 | 8 | 2 |
| RVS035 | 1 | 1 | 1 | 1 | 1 | 1 | 0 | 0 | 0 | 1 | 1 | 1 | 9 | 2 |
| RVS037 | 1 | 1 | 1 | 1 | 1 | 1 | 0 | 1 | 1 | 1 | 1 | 1 | 11 | 3 |
| RVS039 | 1 | 1 | 1 | 1 | 1 | 1 | 0 | 1 | 1 | 0 | 0 | 0 | 8 | 2 |
| RVS041 | 0 | 0 | 1 | 0 | 0 | 1 | 0 | 0 | 0 | 0 | 0 | 1 | 3 | 2 |
| RVS051 | 1 | 1 | 1 | 1 | 1 | 1 | 1 | 1 | 1 | 1 | 1 | 1 | 12 | 3 |
| RVS058 | 1 | 1 | 1 | 1 | 1 | 1 | 1 | 1 | 1 | 1 | 1 | 1 | 12 | 3 |
| RVS059 | 1 | 1 | 1 | 1 | 1 | 1 | 0 | 0 | 0 | 1 | 1 | 1 | 9 | 2 |
| RVS062 | 1 | 1 | 1 | 1 | 1 | 1 | 0 | 0 | 0 | 1 | 1 | 1 | 9 | 2 |
| RVS063 | 1 | 1 | 1 | 1 | 1 | 1 | 0 | 0 | 0 | 1 | 1 | 1 | 9 | 2 |
| RVS072 | 0 | 1 | 1 | 0 | 1 | 1 | 0 | 0 | 0 | 0 | 0 | 0 | 4 | 1 |
| RVS073 | 1 | 1 | 1 | 1 | 1 | 1 | 1 | 1 | 1 | 1 | 1 | 1 | 12 | 3 |
| RVS074 | 1 | 1 | 1 | 1 | 1 | 1 | 0 | 0 | 0 | 1 | 1 | 1 | 9 | 2 |
| RVS076 | 0 | 1 | 1 | 0 | 1 | 1 | 0 | 0 | 0 | 0 | 0 | 0 | 4 | 1 |
| RVS086 | 0 | 1 | 1 | 0 | 1 | 1 | 0 | 0 | 0 | 0 | 1 | 1 | 6 | 2 |
| RVS094 | 1 | 1 | 1 | 1 | 1 | 1 | 1 | 1 | 1 | 1 | 1 | 1 | 12 | 3 |
| RVS098 | 0 | 1 | 1 | 1 | 1 | 1 | 1 | 1 | 1 | 0 | 1 | 1 | 10 | 3 |
| Total | 13 | 20 | 21 | 17 | 21 | 22 | 9 | 11 | 11 | 13 | 17 | 18 | 189 | 49 |

Legend: VR: Vaginorectal Eswab, V: Vaginal Eswab, R: Rectal Eswab, N: No, Y: yes.
